# Risk factors and potential predictors of pulmonary embolism in cancer patients undergoing thoracic and abdominopelvic surgery: a case control study

**DOI:** 10.1186/s12959-022-00442-7

**Published:** 2022-12-22

**Authors:** Yi Li, Zhenjun Liu, Chen Chen, Dan Li, Huan Peng, Pei Zhao, Jiuhui Wang

**Affiliations:** Department of Intensive Care Unit, Sichuan Cancer Hospital & Institute, Surgical Building, No. 55 4th section of South Renmin Road, Chengdu, Sichuan China

**Keywords:** Pulmonary embolism, Thoracic cancer, Abdominopelvic cancer, Risk factors, Predictors

## Abstract

**Background:**

Postoperative pulmonary embolism (PE) is a severe complication leading to death and poor prognosis. The present study investigated the risk factors and potential predictors of PE in cancer patients undergoing thoracic and abdominopelvic surgery.

**Methods:**

A retrospective study was conducted on the patients with cancer who underwent thoracic and abdominopelvic surgery in Sichuan Cancer Hospital from December 2016 to January 2022. A total of 189 patients were included, in which 63 patients diagnosed PE after operation were collected as PE group, and 126 patients matched by age, type of cancer and cancer location were enrolled as control group. Conditional logistic regression was conducted to analyze the association between PE and risk factors. Predictive values of key factors were compared by the area under the curve (AUC) in receiver operating characteristic curve (ROC) curve.

**Results:**

Conditional multivariate logistic regression showed that BMI (odds ratio [OR] 4.065, 95% confidence interval [CI] 1.138–14.527; *p* = 0.031), intraoperative hypotension time (OR 4.095, 95% CI 1.367–12.266; *p* = 0.009), same day fluid balance (OR 0.245, 95% CI 0.061–0.684; *p* = 0.048), and postoperative D-Dimer (OR 1.693, 95% CI 1.098–2.611; *p* = 0.017) were significantly related to the occurrence of postoperative PE. Postoperative D-Dimer had the maximal AUC value 0.8014 (95% CI: 0.7260–0.8770) for predicting PE, with a cutoff value of 1.505 μg/ml.

**Conclusions:**

BMI, intraoperative hypotension time, lower same day fluid balance and postoperative D-dimer are independent risk factors associated with PE in cancer patients undergoing thoracic and abdominopelvic surgery. Postoperative D-Dimer seems to be a good indicator to predict postoperative PE for cancer patients.

Pulmonary embolism (PE) is caused by any embolus blocking pulmonary artery or its branches, and over 90% of emboli come from the floating clots formed in the deep vein of the legs [1]. Deep venous thrombosis (DVT) and PE have the similar provoking risk factors such as advancing age, cancer, trauma, obesity [2] and therefore referred as venous thromboembolism (VTE). A massive pulmonary embolism may induce severe dyspnea, right ventricular dysfunction, cardiogenic shock, circulatory failure in a short time, and is a common cause of sudden death in hospitalized patients.

Among patients with active cancer, the incidence of VTE is 4 to 7 times higher than general population, and the incidence in cancer patients varies greatly by cancer site. The rate of VTE is 3 to 4 times greater in patients with thoracic or abdominal cancer than patients with breast cancer or thyroid cancer, while the highest incidence in pancreatic cancer reaches up to 15% [3, 4]. The most effective therapy for majority cancer patients remains surgical resection, however, VTE as a serious postoperative complication severely influence patients’ recovery and prognosis. Although the risk of VTE was not high compared with other postoperative complications, it is the most common cause of death at 30 days after surgery [5]. The 5-year overall survival of cancer patients with postoperative VTE was proved to be lower than no-VTE cancer patients [6]. The mean incidence of postoperative DVT and PE in patients underwent surgical procedures were 0.2 and 0.08% respectively in China [7]. Meanwhile, hospitalized cancer patients complicated by acute PE is associated with a 90% increase in all-cause mortality, longer length of stay and higher medical costs [8]. The long- term complications of acute PE including chronic thromboembolic pulmonary hypertension and recurrence of PE indicate a poor prognosis.

Besides the well-established risk factors for PE including advanced age, obesity, long-time bed rest, prolonged operation times, malignant tumor is the greatest risk factor for PE in hospitalized patients according to the literature [9]. Furthermore, malignancy-related risk factors such as cancer type, cancer stage and primary site have been documented to be associated with VTE and PE in cancer patients [10, 11]. However, little is known on the risk factor of PE in patients with cancer after surgery. The aim of this study is to investigate the risk factors of PE in patients who undergo surgery for thoracic, abdominal and pelvic cancer besides the common risk factors published in literatures.

## Materials and methods

### Patients

This retrospective study was approved by the Ethics Committee for Medical Research and New Medical Technology of Sichuan Cancer Hospital. The 63 patients with cancer who underwent thoracic and abdominopelvic surgery and diagnosed as postoperative PE at Sichuan Cancer Hospital from December 2016 to January 2022 were collected as PE group, while 126 cancer patients underwent thoracic or abdominopelvic surgery without PE during the same period were assigned in a two-to-one ratio matched by age (± 3 years), type of cancer and cancer location as control group. PE was diagnosed according to ESC (Europe Society Cardiology) guidelines, either by computed tomographic pulmonary angiography (CTPA) or echocardiography after the occurrence of clinical symptoms [12]. The exclusion criteria were as follow: (1) benign neoplasm, (2) younger than 18 years old.

### Data collection

All the demographic and clinic characteristic were retrieved, including: age, gender, weight, Body Mass Index (BMI), cancer stage, histology, cancer location, prolonged bed rest. And past medical history including tobacco abuse, alcohol abuse, hypertension, diabetes, Chronic Obstructive Pulmonary Disease (COPD), atrial fibrillation, hyperlipemia, coronary heart disease, cerebrovascular disease, prior major surgery, and cancer-related treatment such as neoadjuvant chemotherapy, neoadjuvant radiotherapy and immunotherapy before surgeries were collected. The intraoperative data were also recorded, such as operation time, intraoperative hypotension time, type of surgery, blood loss, use of vasoactive drug, arrhythmia. And the postoperative data including postoperative day (POD) 1 fluid volume, POD1 fluid balance, POD2 fluid volume, POD2 fluid balance, severe infection, postoperative atrial fibrillation, postoperative prophylaxis and etc. were collected. Besides, we also retrieved the hematologic tests and biochemical tests parameters including both preoperative and POD1 white blood cell (WBC) count, hemoglobin, platelet (PLT) count, D-Dimer.

Fluid balance was calculated by total input (infusion and by mouth) minus total output (urinary output and drainage volume). Intraoperative hypotension was defined as the mean blood pressure (MBP) less than 70 mmHg, and type of surgery including open surgery and laparoscopy/thoracoscopy. The postoperative prophylaxis contained injection of low-molecular-weight heparin and mechanical prophylaxis including compression stockings and pneumatic compression pump. We also recorded the preoperative and postoperative Caprini scores, a risk scoring system to predict the development of VTE [13].

### Statistical analysis

Data were analyzed and described using STATA 14 software (StataCorp, College Station, TX). Descriptive statistics such as absolute and relative frequencies for discrete parameters and mean, median, standard deviation, and percentiles for continuous parameters were computed. The characteristics of included patients between the PE and non-PE groups were compared using t-test, *x*^2^ test, and Wilcoxon signed-rank test. Statistically significant variables were identified by conditional univariate and multivariate logistic regression via backward elimination method, which included all potential variables and at each step gradually eliminates variables from the regression model to find a reduced model that best explains the data. Comparison of predictive powers of factors was conducted by the area under the curve (AUC) of receiver operating characteristic curve (ROC). Optimal cutoff value was selected based on combined maximum sensitivity and specificity. A *P* value < 0.05 was considered statistically significant.

## Results

### Population description

A total of 189 patients with cancer who underwent surgery were included in this study. Among these patients, the number of males was 101 (53.4%), and for females was 88 (46.6%) and the age ranged from 36 to 83 years. 63 symptomatic patients (30 females and 33 males) diagnosed with PE either by CTPA or echocardiography were included in the PE group, and 126 cases (58 females and 68 males) were included in the non-PE group. There were 129 (68.3%) patients with cancer in thoracic cavity, 60 (31.7%) patients with cancer in abdominopelvic cavity. Adenocarcinoma accounts for the majority histologic type of both groups, 31% in PE group and 70% in non-PE group, respectively. The other comorbidities and cancer-related characteristics of included patients were described in Table 1.

**Table 1:** The baseline characteristics of cancer patients before surgery

Variables	Non-PE group (***N*** = 126)	PE group (***N*** = 63)	***P-value***
**Age (y)**	63 (56–71)	66 (56–71)	0.756
**Gender [No. (%)]**			0.837
Female	58 (46.03)	30 (47.32)	
Male	68 (53.97)	33 (52.38)	
**BMI (kg/m**^**2**^**)**			**< 0.001**
< 22.03	51 (40.48)	12 (19.05)	
22.03–24.69	45 (35.71)	18 (28.57)	
≥ 24.69	30 (23.81)	33 (52.38)	
**Tobacco abuse**	60 (47.62)	27 (42.86)	0.536
**Alcohol abuse**	38 (30.16)	16 (25.40)	0.492
**Hypertension**	27 (21.43)	22 (34.92)	**0.046**
**Diabetes**	17 (13.49)	4 (6.35)	0.141
**COPD**	32 (25.4)	14 (22.22)	0.632
**Atrial fibrillation**	3 (2.38)	2 (3.17)	0.749
**Hyperlipemia**	63 (50.0)	25 (39.68)	0.180
**Coronary heart disease**	3 (2.4)	1 (1.59)	0.715
**Cerebrovascular disease**	1 (0.84)	1 (1.59)	0.646
**Central venous access**	42 (33.33)	21 (33.33)	1.000
**Cancer-related characteristics [No. (%)]**			
**Prior major surgery [No. (%)]**	0 (0.00)	5 (7.94)	**0.001**
**Tumor stage**			0.233
Tis	3 (2.38)	0 (0.0)	0.397
I/mia	32 (25.40)	23 (36.51)	
II	37 (29.37)	16 (25.40)	
III	37 (29.37)	18 (28.57)	
IV	17 (13.49)	6 (9.52)	
**Pathology**			0.882
Adenocarcinoma	70 (55.56)	31 (49.21)	
Squamous cell carcinoma	39 (30.95)	20 (31.75)	
Neuroendocrine carcinoma	7 (5.56)	5 (7.94)	
Sarcoma	3 (2.38)	2 (3.17)	
Others	7 (5.56)	5 (7.94)	
**Cancer location**			0.137
Thoracic cavity	86 (68.3)	43 (68.3)	
Abdominopelvic cavity	40 (31.7)	20 (31.7)	
**Neoadjuvant chemotherapy**	14 (11.11)	7 (11.11)	1.000
**Neoadjuvant radiotherapy**	3 (2.38)	1 (1.59)	0.721
**Preoperative anticoagulation**	11 (8.73)	3 (4.76)	0.326

*BMI* body mass index, *COPD* Chronic Obstructive Pulmonary Disease

### Comparisons of characteristics between PE and non-PE groups

No significant differences in gender were identified between PE and non-PE group. The BMI of 51 (40.48%) patients in non-PE group was smaller than 22.03 kg/m^2^ and 30 (23.81%) patients’ BMI was greater than 24.69 kg/m^2^, while only 12 (19.05%) patients in PE group had smaller BMI and 33 (52.38%) patients’ BMI was greater than 24.69 kg/m^2^. The PE group had a higher BMI than non-PE group (*p* < 0.001). Compared with non-PE group, the difference in the prevalence of hypertension (34.9% vs 17.0%, *p* = 0.046) and proportion of prior major surgery (7.9% vs 0, *p* = 0.001) was significant. There were no significant differences in cancer stage, pathology, preoperative cancer-related therapies or other comorbidities like diabetes, COPD, coronary heart disease and cerebrovascular disease. The results of the comparisons of baseline characteristics of the two groups were shown in Table 1.

The intraoperative hypotension time was significantly longer among patients in PE group than in non-PE group [0 (0–0.5) h vs 0.5 (0–1.42) h, *p* = 0.003)] and blood loss [100 (50–200) ml vs 200 (100–300) ml, *p* = 0.006)]. The PE group had higher proportion of transfusion (20.63% vs 7.14%, *p =* 0.006), a higher incidence of severe infection (6.35% vs 0.79%, *p <* 0.001) and postoperative atrial fibrillation (6.35% vs 0.79%, *p* = 0.025) compared with non-PE group. There was a significantly smaller same day fluid balance [1.08 (0.56–1.54) ml/kg/h vs 1.25 (0.93–1.67) ml, *p* = 0.033)] and lower POD1 fluid volume [1.67 (1.45–2.17) ml/kg/h vs 1.94 (1.57–2.40) ml, *p* = 0.019)] among patients with PE. Besides, the difference in postoperative mechanical prophylaxis rate (84.13% vs 99.21%, *p* < 0.001) and postoperative anticoagulative agent (44.44% vs 64.29%, *p =* 0.009) rate between two groups were significant. (Table 2).

**Table 2:** Perioperative characteristics and laboratory results of the patients underwent surgery

Variables	Non-PE group (***N*** = 126)	PE group (***N*** = 63)	***P-value***
**Operation time (h)**	2.95 (2–3.5)	3.0 (2–4)	0.119
**Intraoperative hypotension time (h)**	0.00 (0.00, 0.50)	0.50 (0.00, 1.42)	**0.003**
**Blood loss (ml)**	100.00 (50.00, 200.00)	200.00 (100.00, 300.00)	**0.006**
**Vasoactive drugs [No. (%)]**	24 (19.05)	19 (30.16)	0.086
**Arrhythmia [No. (%)]**	3 (2.38)	2 (3.17)	0.749
**Same day fluid volume (ml)**	3716 (3046-4384)	3739 (3199-4375)	0.840
**Same day fluid volume (ml/kg/h)**	2.66 (2.18–3.32)	2.43 (2.07–2.98)	0.105
**Same day Fluid balance (ml)**	1765 (1302-2347)	1583 (906–2250)	0.193
**Same day Fluid balance (ml/kg/h)**	1.25 (0.93–1.67)	1.08 (0.56–1.54)	**0.033**
**POD1 Fluid volume (ml)**	2877.5 (2281.5-3289)	2678 (2200-3217)	0.495
**POD1 Fluid volume (ml/kg/h)**	1.94 (1.58–2.40)	1.67 (1.45–2.17)	**0.019**
**POD1 Fluid balance (ml)**	596 (99–1274.5)	576 (− 210–1154)	0.345
**POD1 Fluid balance (ml/kg/h)**	0.44 (0.06–0.96)	0.33 (−0.12–0.79)	0.216
**Open surgery [No. (%)]**	44 (34.92)	29 (46.03)	0.139
**Transfusion [No. (%)]**	9 (7.14)	13 (20.63)	**0.006**
**Severe infection [No. (%)]**	1 (0.79)	19 (30.16)	**< 0.001**
**Postoperative atrial fibrillation [No. (%)]**	1 (0.79)	4 (6.35)	**0.025**
**Postoperative mechanical prophylaxis [No. (%)]**	125 (99.21)	53 (84.13)	**0.000**
**Postoperative anticoagulative agent [No. (%)]**	81 (64.29)	28 (44.44)	**0.009**

*POD* postoperative day

The comparisons of laboratory results between PE and non-PE groups demonstrated that patients in the PE group had higher preoperative D-Dimer [0.32 (0.23, 0.65) μg/ml vs 0.23 (0.19, 0.34) μg/ml, *p* < 0.001], higher postoperative D-Dimer [2.70 (1.85, 4.12) μg/ml vs 0.99 (0.50, 2.12) μg/ml, *p* < 0.001]. There were no statistical differences in preoperative WBC, preoperative hemoglobin, preoperative platelet, postoperative WBC, postoperative hemoglobin, postoperative platelet and Caprini score before and after surgery (Table 3).

**Table 3:** laboratory data and Caprini scores of the patients underwent surgery

Variables	Non-PE group (***N*** = 126)	PE group (***N*** = 63)	***P-value***
Preoperative WBC (10*9/L)	6.09 (5.03–7.12)	5.71 (4.82–6.58)	0.182
**Preoperative Hemoglobin (g/L)**	129 (118–139)	126 (118–140)	0.714
Preoperative platelet (10*9/L)	184.5 (142–227)	180 (152–222)	0.926
**Preoperative D-dimer (μg/ml)**	0.23 (0.19, 0.34)	0.32 (0.23, 0.65)	**< 0.001**
Postoperative WBC (10*9/L)	10.61 (9.05–13.08)	10.01 (7.86 + 13.53)	0.344
**Postoperative Hemoglobin (g/L)**	112 (101–126)	107 (99–123)	0.173
Postoperative platelet (10*9/L)	166.5 (131–209)	166 (131–211)	0.971
**Postoperative D-dimer (μg/ml)**	0.99 (0.50–2.12)	2.70 (1.85, 4.12)	**< 0.001**
**Preoperative Caprini score**	5 (4–6)	5 (4–6)	0.915
**Postoperative Caprini score**	9.13 ± 1.61	9.59 ± 1.94	0.090

*WBC* white blood cell

### Conditional univariate analysis and multivariate logistic regression

We included all variates and showed variates with statistically significance in the conditional univariate analysis and multivariate logistic regression. Results shown in Table 4 demonstrated that BMI tertiles (odds ratio [OR] 4.065, 95% confidence interval [CI] 1.138–14.527; *p* = 0.031), intraoperative hypotension time (OR 4.095, 95% CI 1.367–12.266; *p* = 0.009), same day fluid balance (OR 0.245, 95% CI 0.061–0.684; *p* = 0.048), and postoperative D-Dimer (OR 1.693, 95% CI 1.098–2.611; *p* = 0.017) were significantly related to the incidence of postoperative PE.

**Table 4:** key factors associated with pulmonary embolism by logistic analysis

Variables	Univariate Analysis			Multivariate Analysis		
	OR	95%CI	***P*** value	OR	95%CI	***P*** value
**Hyperlipemia**	0.436	0.195–0.975	0.043			
**BMI tertiles**	3.095	1.815–5.279	< 0.001	4.065	1.138–14.527	0.031
**Diabetes**	0.233	0.061–0.898	0.034			
**Intraoperative hypotension time**	3.257	1.419–7.473	0.005	4.095	1.367–12.266	0.009
**Same day fluid balance (ml/kg/h)**	0.354	0.139–0.899	0.029	0.245	0.061–0.684	0.048
**POD1 Fluid balance (ml/kg/h)**	0.727	0.513–1.032	0.074			
**Postoperative D-dimer**	1.637	1.109–2.516	0.013	1.693	1.098–2.611	0.017
**Postoperative anticoagulative agent**	0.171	0.050–0.582	0.005			
**Postoperative mechanical prophylaxis**	0.073	0.006–0.938	0.045			

*BMI* body mass index, *POD* postoperative day

**Fig. 1:**
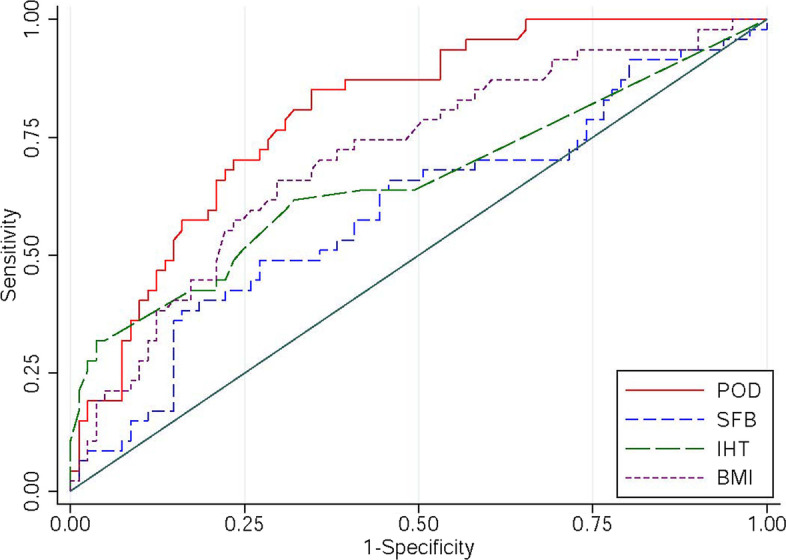
ROC curves of potential predictive variates for postoperative PE (POD: postoperative D-dimer, SFB: same day fluid balance, IHT: intraoperative hypotension time, BMI: body mass index)

We further plotted corresponding ROC curves to illustrate predictive value of the four significant variates according to multivariate logistic regression. ROC-AUC was 0.8014 [0.7259–0.8770] in postoperative D-dimer, 0.7094 [0.6157–0.8030] in BMI, 0.6567 [0.5557–0.7577] in intraoperative hypotension time, 0.5965 [0.4913–0.7017] in same day fluid balance. The optimal cutoff values of postoperative D-dimer were 1.505 μg/ml to predict postoperative PE (Fig. 1).

## Discussion

Malignant tumor patients are often at a high risk of PE. A study reported that a 3-fold incidence of fatal PE and 4-fold perioperative mortality among cancer patients than among non-cancer patients after surgery [14]. The main treatments of PE include haemodynamic and respiratory support, initial anticoagulation and reperfusion treatment according to the recommendation of the guidelines [15]. However, recent major surgery is a relative contraindication of anticoagulation and thrombolytic, which may significantly increase the risk of extensive incision bleeding and therefore brings huge difficulty in the treatment of postoperative PE and poor prognosis of these patients.

As a part of VTE, PE may have the same pathogenesis of VTE described as Virchow’s triad including prolonged immobility, venous stasis and vascular endothelial injury [16]. Besides, cancer cell can active coagulation pathway by producing either tissue factor and other coagulative factors directly or cytokines that triggers immune system and platelets indirectly [17]. Moreover, the direct injury of tissue and vascular endothelial by surgery active the coagulation system further. There are many risk scoring models for evaluating the risk of VTE and PE. We used Caprini risk scoring model which included factors associated with operation to assess two groups of patients. However, there was no significant statistical difference in Caprini scores before and after surgery between two groups, so we inferred there may be other risk factors not included in this scoring model.

The results of univariate and conditional multivariate logistic regression showed higher BMI was an independent risk factor associated with PE. Overweight patients may have an increased risk of PE as a result of endothelial injury and dysfunction caused by inflammatory cytokine associated with obesity [18]. Although the relationship between BMI and VTE has been well established by many studies [2, 19], current literature remains controversial on the impact of BMI in risk of PE. A retrospective study found out that increased BMI and obesity were risk factors for the development of PE and VTE in patients undergoing pancreatic surgery [20], while a large database study pointed out that overweight was a risk factor only of development of PE instead of DVT after primary total hip and knee arthroplasty [21]. The BMI threshold was varied in different studies, the median BMI of patients with PE reported by Mussle et al. [20] was significantly higher that BMI of patients with no PE (28.1 kg/m^2^ vs. 24.8 kg/m^2)^ and Sloan et al. [21] found the risk of PE was significantly elevated in patients with BMI greater than 35 kg/m^2^. The cutoff value of BMI in our study was 24.40 kg/m^2^ which was lower than many findings reported. These results may be due to the underweight of cancer patients who suffer from malnutrition and cachexia for long time. Therefore, the BMI threshold of cancer patients may need further investigation.

Besides, we also found significant statistic differences in hypertension ratio and intraoperative hypotension time between the two groups. Although no significant statistic difference found in hypertension from multivariate logistic regression, a recent published study pointed invasive cancer, chronic hypertension, cancer type, and evidence of metastasis were the most significant risk factors for VTE in women who underwent breast surgery [22]. And a study aimed to assess the relationship between cardiovascular disease and the occurrence of VTE found that hypertension increased the risk of VTE in patients with newly diagnosed lung cancer, which may be mediated by inflammation [23]. PE group had longer intraoperative hypotension time compared to non-PE group, and the results of conditional multivariate logistic regression indicated intraoperative hypotension time was an independent risk factor associated with postoperative PE. Blood pressure of patients who underwent surgery may decrease in different levels because of several reasons: relative hypovolemia due to preoperative fasting and intraoperative blood loss, decreasing of cardiac output led by the use of anaesthetics, sedatives, analgesics during general anesthesia which may have negative inotropic effect on myocardial contraction, the decrease of peripheral resistance caused by muscle relaxant and controlled hypotension to avoid severe bleeding. The mechanism of increased risk of VTE and PE led by hypotension was unclear. It might be related to venous stasis and the inflammation trigged by insufficient tissue perfusion after blood pressure fell. There is rare study evaluating the relationship between intraoperative hypotension time and PE. A study pointed out there was a positive association between orthostatic hypotension status and incident VTE, which may be explained by long duration of blood pooling [24]. Another group assessed risk factors associated with postoperative PE after radical resection of head and neck cancers also found cumulative duration of intraoperative hypotension may increase the incidence of PE [25]. The relationship between the level of decline in blood pressure and duration of intraoperative hypotension time and postoperative PE needs further investigation.

Our analysis identified same day fluid balance was related to the risk of occurrence of postoperative PE. The same day fluid balance was 1765 (1302-2347) ml in non-PE group and was 1583 (906–2250) ml in PE group. And we convert the unit of account of fluid balance to “ml/kg/h” to eliminate impact brought by bodyweight. The results of multivariate logistic regression showed same day fluid balance was a protective factor of the incidence of postoperative PE. There are many studies focusing on the relationship between different intraoperative fluid infusion volume and the incidence of postoperative complications. Two studies including patients undergoing minimally invasive lobectomy [26] and open thoracotomy [27] figured out that both restrictive and liberal intraoperative fluid administration were related to adverse effects on postoperative outcomes. Meanwhile, a recent study reported a greater intraoperative positive fluid balance was independently associated with a higher incidence of complications including VTE [28]. Moreover, the fluid volume varied greatly in different studies, Kim et al. [27] study suggested intraoperative net fluid infusion at 4–5 ml/kg/h with better results while Wu et al. [26] suggested moderate fluid infusion at greater than 9.4 ml/kg/h and less than 11.8 ml/kg/h. However, these studies only assessed the intraoperative fluid strategies, very few study paid attention to same day fluid administration. Our results suggested fluid management should be supervised not only during the operation, but also during the same day and appropriate fluid balance maybe better than restrictive fluid strategies.

Plasma D-dimer is a specific marker of hypercoagulation and secondary fibrinolytic activity, therefore, it is widely used for excluding VTE with the threshold as 500 ng/ml since its high prognostic sensitivity [29]. Since plasma D-dimer level increases with age, tumor, surgery, infection and some other disease status, age-adjusted D-dimer cutoff levels were reported to help to rule out PE among cancer patients [30, 31]. However, the D-dimer predictive value of PE remains controversial. According to our results, preoperative D-dimer in two groups were significantly different. The upper quartile of preoperative D-dimer level was greater than 0.63 μg/ml, that was higher than the normal threshold. A Japanese study included patients with urologic malignancy pointed that elevated D-dimer (> 0.5 μg/ml) prior to surgery was a predictors for deep venous thrombosis [32]. And a Chinese team constructed a risk assessment model for VTE after colorectal cancer surgery including preoperative D-dimer (> 0.49 μg/ml) as a factor of four [33]. Based on our multivariate logistic analysis we found postoperative D-dimer an independent risk factor of PE. The postoperative D-dimer level in PE group was 2.70 (1.85, 4.12) μg/ml, and 0.99 (0.50, 2.12) μg/ml in non-PE group. Postoperative plasma D-dimer level can increase apparently from baseline, and was reported vary from 2.563 μg/ml to 4.125 μg/ml [34, 35]. Hence it is difficult to figure out the cutoff of D-dimer value or the raising rate to predict a possible PE. Another study suggested continuous detection of D-dimer levels after pulmonary tumor surgery [36], we did observe D-dimer levels keep raising after operation in PE group, but some of patients in PE group were soon diagnosed PE within 24 hours or 48 hours after surgery which made it impossible for further data collection. We recommend continuous monitor plasma D-dimer according to the results of logistic analysis and possible Doppler or CTPA may be arranged if D-dimer levels keep rising.

## Limitations

Our study was based on the retrospective analysis of a small sample data from one single medical center. We could not extract variable like first ambulation time after operation since these data were not routinely recorded in the medical notes. Besides, some asymptomatic PE patients were missed because they were undiagnosed. We suspect postoperative severe infection is an independent risk factor, and the univariate analysis indicated postoperative severe infection may relate to PE, however the small number of patients with severe infection did not allow further analysis. Therefore, the prospective large sample studies need to be conducted to further identify our findings.

## Conclusions

In summary, cancer patients underwent surgery are at a high risk of postoperative PE. BMI, intraoperative hypotension time, lower same day fluid balance and postoperative D-dimer are independent risk factors associated with postoperative PE in patients with thoracic and abdominopelvic cancer. Further larger sample size prospective sturdies are required to confirm our data.

## Data Availability

The datasets used and/or analyzed during the current study are available from the corresponding author on reasonable request.
